# Progress Toward Measles Elimination — Pakistan, 2000–2018

**DOI:** 10.15585/mmwr.mm6822a4

**Published:** 2019-06-07

**Authors:** Mohammed Osama Mere, James L. Goodson, Arshad K. Chandio, Muhammad Suleman Rana, Quamrul Hasan, Nadia Teleb, James P. Alexander

**Affiliations:** ^1^Communicable Diseases Cluster, Pakistan Country Office, World Health Organization; ^2^Global Immunization Division, Center for Global Health, CDC; ^3^National Immunization Program, Federal Ministry of Health, Islamabad, Pakistan; ^4^National Institutes for Health, Islamabad, Pakistan; ^5^Vaccine Preventable Diseases and Immunization, World Health Organization Eastern Mediterranean Regional Office, Cairo, Egypt.

In 1997, the 21 countries in the World Health Organization (WHO) Eastern Mediterranean Region* (EMR) passed a resolution during the 41st session of the Regional Committee for the Eastern Mediterranean to eliminate measles^†^ (*1*). In 2015, this goal was included as a priority in the Eastern Mediterranean Vaccine Action Plan 2016–2020 (*2*), approved at the 62nd session of the Regional Committee (*3*). To achieve measles elimination, the WHO Regional Office for the Eastern Mediterranean developed the following four-pronged strategy: 1) achieve ≥95% vaccination coverage with the first dose of measles-containing vaccine (MCV) among children in every district of each country through routine immunization services; 2) achieve ≥95% vaccination coverage with a second MCV dose in every district of each country either through implementation of a routine 2-dose vaccination schedule or through supplementary immunization activities (SIAs)^§^; 3) conduct high-quality, case-based measles surveillance in all countries; and 4) provide optimal measles clinical case management, including dietary supplementation with vitamin A (*4*). Pakistan, an EMR country with a population of approximately 200 million, accounts for nearly one third of the overall EMR population. This report describes progress and challenges toward measles elimination in Pakistan during 2000–2018. During the study period, estimated coverage with the first MCV dose (MCV1) increased from 57% in 2000 to 76% in 2017. The second MCV dose (MCV2) was introduced nationwide in 2009, and MCV2 coverage increased from 30% in 2009 to 45% in 2017. During 2000–2018, approximately 232.5 million children received doses of MCV during SIAs. Reported confirmed measles incidence increased from an average of 24.6 per 1 million persons during 2000–2009 to an average of 80.4 during 2010–2018, with peaks in 2013 (230.3) and 2018 (153.6). In 2017 and 2018, the rates of suspected cases discarded as nonmeasles after investigation were 2.1 and 1.5 per 100,000 population, reflecting underreporting of cases. To achieve measles elimination, additional efforts are needed to increase MCV1 and MCV2 coverage, develop strategies to identify and reach communities not accessing immunization services, and increase sensitivity of case-based measles surveillance in all districts.

## Immunization Activities

MCV1 was introduced in the routine childhood immunization schedule nationwide in Pakistan in 1974 (*4*), and MCV2 was added to the schedule in 2009. The doses are administered to children at ages 9 and 15 months. Administrative vaccination coverage^¶^ data are reported each year from all districts** in Pakistan to the National Immunization Programme, where they are aggregated and reported to WHO and the United Nations Children’s Fund (UNICEF) through the Joint Reporting Form. WHO and UNICEF use reported administrative coverage and available survey results to generate annual estimates of vaccination coverage through routine immunization services (*5*). Estimated MCV1 coverage in Pakistan increased from 57% in 2000 to 76% in 2017, and estimated MCV2 coverage increased from 30% in 2009 to 45% in 2017 (Figure). A Demographic and Health Survey implemented nationwide during 2017–2018 estimated MCV1 and MCV2 coverage at 73% and 67%, respectively. Among the eight provinces and federal areas, survey estimates of MCV1 and MCV2 coverage were highest in Punjab (85% and 82%, respectively), Islamabad (83%, 77%), and Azad Jammu and Kashmir (83%, 75%); intermediate in Gilgit-Baltistan (66%, 62%), Khyber Pakhtunkhwa (63%, 50%) and Sindh (61%, 60%); and lowest in the Federally Administered Tribal Areas (35%, 21%) and Balochistan (33%, 34%) (*6*).

**FIGURE:**
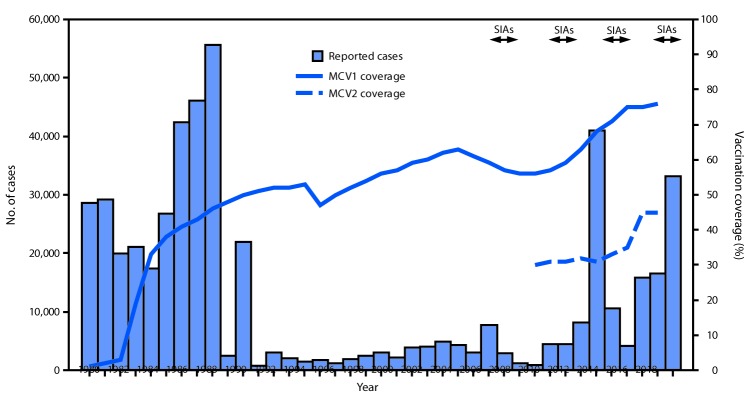
Number of reported measles cases and estimated coverage with the first and second doses of measles-containing vaccine (MCV), and supplemental immunization activities (SIAs), by year* — Pakistan, 1980–2018 **Abbreviations:** MCV1 = first dose of measles-containing vaccine; MCV2 = second dose of measles-containing vaccine. * For 1980–2012, cases were reported through the World Health Organization and United Nations Children’s Fund Joint Reporting Form. For 2013–2018, cases were reported through the national case-based measles surveillance system.

During 2005–2018, approximately 232.5 million children received MCV during SIAs (Table 1). A nationwide catch-up SIA was conducted in five phases during 2007–2008 and reached 66.6 million children aged <15 years with >100% administrative coverage documented. Following extensive flooding in the Indus River Basin in 2010, affecting much of Khyber Pakhtunkhwa, Punjab, and Sindh Provinces, subnational measles SIAs were conducted during 2010–2011 for children aged <13 years in flood-affected areas and aged <5 years in other areas; 29.7 million children were vaccinated (94% administrative coverage). In response to a measles epidemic in 2013, a nationwide SIA was conducted in phases during 2014–2015, and 61.4 million children aged <10 years were vaccinated (103% administrative coverage). An independent post-SIA coverage survey conducted in Sindh Province estimated 83% coverage. In response to a measles epidemic during 2017–2018, a nationwide SIA was conducted in 2018, and 37.1 million children aged <5 years (children aged <7 years in Punjab Province) were vaccinated; an independent post-SIA coverage survey estimated that SIA coverage was 93.3% overall and 95.7% in Punjab. Monovalent measles vaccine was used in all SIAs.

**TABLE 1:** Characteristics of measles supplementary immunization activities (SIAs)* — Pakistan, 2005–2018

Year	Age group targeted	Extent of SIA	Population reached in targeted age group, no. (%)^†^	Vaccination coverage estimate (%)
2005	12–59 mos	Subnational	1,232,000 (77)	—
2007	9 mos–15 yrs	National^§^	2,511,837 (98)	—
	9 mos–13 yrs	National^§^	1,282,232 (105)	—
	9 mos–13 yrs	National^§^	6,906,376 (100)	—
	9 mos–13 yrs	National^§^	20,566,497 (97)	—
2008	9 mos–13 yrs	National^§^	35,315,375 (103)	—
2010	9 mos–13 yrs	Subnational	13,740,906 (90)	—
	6–59 mos	Subnational	6,991,065 (95)	—
	6–59 mos	Subnational	1,007,195 (102)	—
2011	9–59 mos	Subnational	1,492,278 (106)	—
	9–59 mos	Subnational	4,849,193 (94)	—
	9–59 mos	Subnational	919,528 (105)	—
	9–59 mos	Subnational	167,678 (74)	—
	9–59 mos	Subnational	557,681 (98)	—
2012	9 mos–9 yrs	Subnational	1,954,175 (102)	—
2013	9 mos–9 yrs	Subnational	4,002,154 (108)	—
	6 mos–9 yrs	Subnational	26,986,015 (96)	—
2014	6 mos–9 yrs	National^§^	14,026,013 (105)	83 (Sindh Province)
	6 mos–9 yrs	National^§^	9,432,492 (101)	—
	6 mos–9 yrs	National^§^	1,439,892 (100)	—
2015	6 mos–10 yrs	National^§^	30,633,406 (103)	—
	6 mos–10 yrs	National^§^	227,762 (95)	—
	6 mos–10 yrs	National^§^	204,308 (124)	—
	6 mos–10 yrs	National^§^	3,512,771 (101)	—
	6 mos–10 yrs	National^§^	413,695 (100)	—
	6 mos–10 yrs	National^§^	1,519,242 (95)	—
2017	9–59 mos	Subnational	1,302,642 (96)	—
	9–119 mos	Subnational	144,129 (68)	—
	9–59 mos	Subnational	1,034,871 (84)	—
2018	9–119 mos	Subnational	91,111 (99)	—
	6–59 mos	Subnational	914,058 (87)	—
	9–59 mos	National	37,131,234 (105)	93
**2005–2018**			**232,509,811 (100)^¶^**	—

* SIAs generally are carried out using two approaches. An initial, nationwide catch-up SIA targets all children aged 9 months–14 years; it has the goal of eliminating susceptibility to measles in the general population. Periodic follow-up SIAs then target all children born since the last SIA. Follow-up SIAs generally are conducted nationwide every 2–4 years and target children aged 9–59 months; their goal is to eliminate any measles susceptibility that has developed in recent birth cohorts and to protect children who did not respond to the first vaccine dose. The exact age range for follow-up SIAs depends on the age-specific incidence of measles, measles vaccination coverage through routine services, and the time since the last SIA. Monovalent measles vaccine was used in all SIAs.

^†^ Values >100% indicate that the number of doses administered exceeded the estimated target population.

^§^ Rollover national campaigns started the previous year or will continue into the next year.

^¶^ Average SIA coverage, weighted by size of target population.

## Surveillance Activities and Measles Incidence

Aggregated measles cases^††^ are reported by all health facilities in Pakistan through the National Health Management Information System and reported annually through the Joint Reporting Form. In 2009, case-based measles surveillance^§§^ was initiated in Pakistan following WHO Regional Office for the Eastern Mediterranean guidelines and using the existing vaccine-preventable diseases surveillance system with some modification (*7*). During 2013–2018, the case-based surveillance system was expanded to include additional health facilities; as of 2018, there were 7,555 reporting units. WHO technical officers were appointed in every province and area in the country during 2017–2018 to monitor key surveillance performance indicators.^¶¶^ Reporting of measles virus genotyping to the WHO global measles nucleotide surveillance database was begun in 2007 (*8*).

After implementing nationwide catch-up measles SIAs during 2007–2008, the number of confirmed measles cases decreased from 7,641 in 2006 to 863 in 2009 (Figure). Following extensive flooding and large-scale population movements in 2010, the number of measles cases increased approximately eightfold, from 4,321 in 2010 to 40,923 in 2013, corresponding to an incidence of 230.3 per million. Following SIAs during 2013–2014, the number of confirmed cases declined to 4,112 in 2015, but increased to 33,007 in 2018 (incidence = 153.6 per million); the majority of these cases occurred before the nationwide SIA conducted in October 2018 (Figure) (Table 2). Overall, measles incidence averaged 24.6 cases per million population during 2000–2009, and 80.4 per million during 2010–2018.

**TABLE 2:** Reported measles incidence, number of measles cases by case classification, age group, and vaccination status based on measles case-based surveillance — Pakistan, 2013–2018

Characteristic	2013	2014	2015	2016	2017	2018
**Reported measles cases and incidence**						
No. of confirmed measles cases	40,923	10,427	4,112	15,791	16,385	33,007
Confirmed measles incidence (cases per 1 million population)	230.3	56.9	22.0	82.8	78.9	153.6
**No. of measles cases by case classification**						
Suspected*	44,586	11,980	5,947	19,147	21,087	36,223
Laboratory-confirmed	8,749	1,409	386	2,703	6,963	4,172
Epidemiologically linked^†^	0	0	0	0	0	3,366
Clinically compatible^§^	32,174	9,018	3,726	13,088	9,422	25,469
Discarded^¶^	3,663	1,553	1,835	3,356	4,702	3,216
**Age group of patients with laboratory-confirmed and epidemiologically linked measles cases, no. (%)**						
<9 mos	—	—	—	—	677 (10)	1,025 (14)
9 mos–4 yrs	—	—	—	—	3,549 (51)	3,805 (50)
5–9 yrs	—	—	—	—	1,441 (21)	1,903 (25)
10–14 yrs	—	—	—	—	200 (3)	281 (4)
≥15 yrs	—	—	—	—	256 (4)	195 (3)
Unknown/Missing	—	—	—	—	840 (12)	329 (4)
**MCV doses received by laboratory-confirmed and epidemiologically linked measles cases, no. (%)**						
≥2	—	—	—	—	781 (11)	621 (8)
1	—	—	—	—	1,083 (16)	685 (9)
0	—	—	—	—	3,777 (54)	2,389 (32)
Unknown	—	—	—	—	482 (7)	453 (6)
Missing	—	—	—	—	840 (12)	3,390 (45)
**Surveillance performance indicators**						
No. of discarded nonmeasles cases per 100,000 population, national level (target: ≥2)	—	—	—	—	2.1	1.5
% of suspected measles cases adequately investigated** within 48 hrs of notification (target: ≥80)	—	—	—	—	0	10
% of suspected measles cases with adequate specimens^††^ tested for measles in a proficient laboratory^§§^ (target: ≥80)	—	—	—	—	54	19
% of results reported by laboratory within 4 days of specimen receipt (target: ≥80)	—	—	—	—	21	11
% of weekly surveillance units reporting to national level on time (target: ≥80)	—	—	—	—	85	100

**Abbreviation:** MCV = measles-containing vaccine.

* An illness in any person a clinician suspects of having a measles infection, or in any person with fever and rash, and cough, coryza or conjunctivitis.

^†^ Epidemiologically linked measles cases are those that occurred in geographic and temporal proximity to a laboratory-confirmed case or to another epidemiologically linked case.

^§^ Clinically compatible measles cases are suspected cases for which there is no laboratory confirmation or epidemiologic link.

^¶^ Discarded nonmeasles cases include those suspected measles cases with an adequate specimen for laboratory testing that were found to be measles immunoglobulin M (IgM) antibody negative or rubella IgM antibody positive.

** Includes collection of all the following data elements regarding each suspected case of measles: patient name or identifiers, place of residence, place of infection (at least to district level), age (or date of birth), sex, date of rash onset, date of specimen collection, measles vaccination status, date of last measles vaccination, date of notification, date of investigation, and travel history.

^††^ Blood specimen collected within 28 days of rash onset.

^§§^ A World Health Organization-accredited laboratory that has an established quality assurance program or one with oversight by a World Health Organization-accredited laboratory.

During 2017 and 2018, the rates of suspected cases discarded after investigation were 2.1 and 1.5 per 100,000 population, respectively (Table 2). During 2007–2018, measles virus genotype results were obtained for 201 confirmed measles cases (50 D4, 150 B3, and 1 H1). D4 genotypes were found during 2007–2013, and B3 genotypes predominated during 2011–2018 with spread of B3 globally during 2010–2018.

## Discussion

During 2000–2017, MCV1 and MCV2 coverage in Pakistan increased substantially, to 76% and 45%, respectively, but remained well below the WHO-recommended level of ≥95%. In addition, large-scale measles outbreaks occurred during 2012–2014 and 2016–2018, revealing coverage gaps from both routine immunization services and SIAs. The 2017–2018 Demographic and Health Survey found that coverage with all basic vaccines (1 dose of Bacille Calmette-Guérin [BCG] vaccine, 3 doses of diphtheria and tetanus toxoids and pertussis [DTP] vaccine, 3 doses of polio vaccine, and 1 dose of measles vaccine) ranged from 80% among children in the highest wealth quintile to 38% among children in the poorest wealth quintile and from 71% among children residing in urban areas to 63% among those in rural areas (*6*). To reduce disparities, increase vaccination coverage, and achieve measles elimination, enhanced efforts are needed to reach all children, particularly those in rural areas and poor communities. Periodic high-quality SIAs conducted according to WHO SIA guidelines, using the WHO SIA readiness assessment tool to ensure ≥95% 2-dose coverage, will require availability of adequate resources for success. For the 2018 SIA, Gavi, the Vaccine Alliance, provided funding support, and WHO, UNICEF and other international partners contributed to SIA planning, implementation, and monitoring. A postcampaign survey documented 93.3% coverage nationally, demonstrating the potential impact that appropriately funded and well-executed activities can have on improving SIA quality.

Case-based measles surveillance was introduced in 2009 and strengthened during 2017–2018. Some of the apparent increase in measles cases, especially during 2013–2018, reflects improved surveillance sensitivity. Nonetheless, WHO standard surveillance indicators reflected underreporting and low sensitivity of case detection overall. To increase case-based surveillance sensitivity to achieve measles elimination, case-based surveillance reporting sites need to be expanded to all health facilities in the country. High-quality nationwide case-based surveillance data are essential for identifying subpopulations with measles susceptibility in need of SIAs.

Pakistan remains one of only three countries worldwide that has never interrupted wild poliovirus type 1 transmission (*9*); therefore, polio eradication activities remain intense in the country. Measles elimination efforts can leverage the polio assets, experience, and capacity to identify and reach communities not accessing routine immunization services; engage local leaders and community members to ensure that all children in the target age groups participate in SIAs; use epidemiologic investigations to identify areas that need additional SIAs; and improve outbreak preparedness and response to rapidly contain outbreaks.

The Eastern Mediterranean Regional Technical Advisory Group on Immunization (RTAG) recommended forming a multipartner taskforce to apply lessons learned from the polio eradication initiative to address gaps in measles vaccination coverage. These include mapping areas where children missed by routine immunization services reside, identifying reasons for being missed, and developing a strategic plan that includes allocation of necessary resources for implementation (*10*). RTAG also recommended introduction of rubella-containing vaccine into the national infant immunization schedule by 2020. Introduction of combined measles-rubella vaccine would provide an opportunity to build population measles immunity to achieve measles and rubella elimination through a measles-rubella vaccine SIA targeting children aged <15 years.

The findings in this report are subject to at least three limitations. First, administrative coverage might overestimate vaccination coverage through erroneous inclusion of SIA doses or doses administered to children outside of target age groups, inaccurate estimates of the target population size, and inaccurate reports of the number of doses delivered. Second, surveillance data likely underestimate measles incidence because not all patients seek care and not all measles patients who seek care are reported. Finally, efforts to strengthen surveillance over time likely led to reporting bias through increased reporting efficiency annually.

To advance progress toward measles elimination in Pakistan, there is a need to raise the visibility of measles elimination efforts, including the benefits of achieving measles elimination. Without jeopardizing the focused efforts to interrupt poliovirus transmission, transitioning the substantial polio infrastructure and resources should be carefully managed to support measles elimination and broader EMR vaccination goals.

**Summary**

**What is already known about this topic?**

In the 2 decades before 2000, estimated coverage with the first measles-containing vaccine dose (MCV1) in Pakistan was ≤57%. The number of reported measles cases per year averaged approximately 29,000 during 1980–1989 and 3,900 during 1990–1999.

**What is added by this report?**

Estimated MCV1 coverage increased from 57% to 76% during 2000–2017, and second-dose coverage increased from 30% to 45% during 2009–2017. Approximately 232.5 million children were vaccinated with MCV during 2005–2018 vaccination campaigns. Despite these efforts, MCV coverage remained well below the recommended level of 95%, and measles incidence increased during 2010–2018.

**What are the implications for public health practice?**

To achieve measles elimination, efforts are needed to increase 2-dose vaccine coverage, reach communities not accessing immunization services, and increase measles surveillance sensitivity.

## Footnotes

* The Eastern Mediterranean Region, one of six regions of the World Health Organization, consists of 21 member states and Palestine (West Bank and Gaza Strip), with a population of nearly 583 million persons. The member states are as follows: Afghanistan, Bahrain, Djibouti, Egypt, Iran, Iraq, Jordan, Kuwait, Lebanon, Libya, Morocco, Oman, Pakistan, Qatar, Saudi Arabia, Somalia, Sudan, Syria, Tunisia, United Arab Emirates, and Yemen.

† Measles elimination is defined as the absence of endemic measles cases for a period of ≥12 months, in the presence of adequate surveillance.

§ SIAs are immunization campaigns, typically carried out using two targeted age ranges. An initial, nationwide catch-up SIA targets all children aged 9 months–14 years, with the goal of eliminating measles susceptibility in the population. Periodic follow-up SIAs then target all children born since the last SIA. Follow-up SIAs generally are conducted every 2 to 4 years and target children aged 9–59 months; the goal of a follow-up SIA is to eliminate any measles susceptibility that has accumulated in recent birth cohorts and to protect children who did not respond to the first dose of measles vaccine.

¶ Administrative vaccination coverage is the number of vaccine doses administered divided by the estimated target population.

** The total number of districts in Pakistan was 147 during 2013–2015 and 151 during 2016–2018.

†† Aggregate measles surveillance involves a report of a summary of suspected measles cases, by age group and location (district), but does not include a line-listing of individual cases.

§§ Case-based measles surveillance includes individual case investigation and blood specimen collection for laboratory testing. Essential data elements to be obtained during the investigation include name or identifier, date of birth or age, sex, place of residence, vaccination status or date of last vaccination, date of rash onset, date of notification, date of investigation, date of specimen collection, and place of infection or travel history.

¶¶ Key surveillance performance indicators include 1) two or more discarded nonmeasles cases per 100,000 population at the national level per year; 2) two or more discarded nonmeasles cases per 100,000 per year in ≥80% of subnational administrative units; 3) adequate investigation of ≥80% of suspected measles cases conducted within 48 hours of notification; 4) adequate collection and testing in a proficient laboratory of specimens from ≥80% of suspected cases for detecting acute measles and rubella infection; 5) receipt of ≥80% of specimens at the laboratory within 5 days of collection; 6) report of ≥80% of serology results by the laboratory within 4 days of specimen receipt; and 7) on-time reporting of measles and rubella data to the national level by ≥80% of surveillance units.
